# Food allergy enhances allergic asthma in mice

**DOI:** 10.1186/s12931-014-0142-x

**Published:** 2014-11-30

**Authors:** Tiphaine Bihouée, Gregory Bouchaud, Julie Chesné, David Lair, Camille Rolland-Debord, Faouzi Braza, Marie-Aude Cheminant, Philippe Aubert, Guillaume Mahay, Christine Sagan, Michel Neunlist, Sophie Brouard, Marie Bodinier, Antoine Magnan

**Affiliations:** INSERM U1087, l’institut du Thorax CHU Nantes, Hôpital Laënnec, 44093 Nantes Cedex 1, France; CNRS, UMR 6291, Nantes, F-44000 France; Université de Nantes, Nantes, F-44000 France; CHU de Nantes, l’institut du thorax, Service de Pneumologie, Nantes, F-44000 France; DHU2020 médecine personnalisée des maladies chroniques, Nantes, F-44000 France; INRA, UR1268 BIA, Nantes, F-44316 France; INSERM, UMR U913, Nantes, F-44000 France; INSERM, UMR U1064 and Institut de Transplantation Urologie, Néphrologie (ITUN), Nantes, F44093 France; CHU de Nantes, Service d’anatomie et cytologique pathologiques, Nantes, France; CHU de Nantes, Service de Pédiatrie, Nantes, F-44000 France

**Keywords:** Asthma, Allergy, Th2 cytokines, Gut, House dust mite, Immunity, Mouse, Ovalbumin

## Abstract

**Background:**

Atopic march refers to the typical transition from a food allergy in early childhood to allergic asthma in older children and adults. However the precise interplay of events involving gut, skin and pulmonary inflammation in this process is not completely understood.

**Objectives:**

To develop a mouse model of mixed food and respiratory allergy mimicking the atopic march and better understand the impact of food allergies on asthma.

**Methods:**

Food allergy to ovalbumin (OVA) was induced through intra-peritoneal sensitization and intra-gastric challenge, and/or a respiratory allergy to house dust mite (HDM) was obtained through percutaneous sensitization and intra-nasal challenges with *dermatophagoides farinae* (Der f) extract. Digestive, respiratory and systemic parameters were analyzed.

**Results:**

OVA-mediated gut allergy was associated with an increase in jejunum permeability, and a worsening of Der f-induced asthma with stronger airway hyperresponsiveness and pulmonary cell infiltration, notably eosinophils. There was overproduction of the pro-eosinophil chemokine RANTES in broncho-alveolar lavages associated with an enhanced Th2 cytokine secretion and increased total and Der f-specific IgE when the two allergies were present. Both AHR and lung inflammation increased after a second pulmonary challenge.

**Conclusion:**

Gut sensitization to OVA amplifies Der f-induced asthma in mice.

## Background

Asthma, a common chronic respiratory disease, affects nearly 300 million people worldwide, and its prevalence exceeds 10% of the population in many industrialized countries [1]. Airway obstruction in asthma and the resulting symptoms are caused by a combination of airway smooth muscle constriction and inflammation of the bronchi [2]. Among the various phenotypes of asthma, early-onset atopic Th2 [3] type is commonly associated with allergic disorders and appears to be the final stage of an “atopic march” from skin or intestinal inflammation leading to sensitization to aeroallergens, and progressing to bronchial asthma [4–6]. Although some of the risk factors of atopic asthma are well known [7], the precise interplay of events involving gut, skin and pulmonary inflammation is not completely understood. It has been suggested that a previous food allergy could increase Th2 inflammation and amplify the asthmatic phenotype in humans, which would give evidence for a link between food allergy and allergic asthma [8,9].

Ovalbumin (OVA) is a frequent food allergen in egg-induced anaphylaxis and atopic dermatitis (AD) in childhood. House dust mites (HDM) [10] are also strongly involved in AD, in which skin disruption facilitates allergen penetration, allergic sensitization and eczema [11,12]. Overall HDM is recognized as the main allergen for atopic asthma [1]. OVA and HDM are therefore highly relevant to the atopic march, inducing food allergy, skin sensitization, and asthma. OVA- or HDM-based allergic models induce eosinophil- and/or neutrophil-based bronchial inflammation according to administration routes, adjuvants and doses of allergens used [13–15]. Yet, none of these models completely explains the atopic march, from intestinal or skin sensitization to asthma.

In the present study, we establish a model of *Dermatophagoides farinae* (Der f)-induced asthma with primary OVA-induced food allergy. The choice of these allergens was driven by their relevance in clinics, with OVA being a major food allergen in early childhood, involved in AD, and Der f a major allergen of HDM, largely involved in AD, allergic rhinitis and asthma in late childhood and adults (1, 10). We demonstrate that food sensitization to one allergen primes the immune system to build an amplified pulmonary inflammation associated with an exaggerated immune reaction in response to another, unrelated allergen.

## Methods

### Induction of food, respiratory, and combined food/respiratory allergy

**Figure 1:**
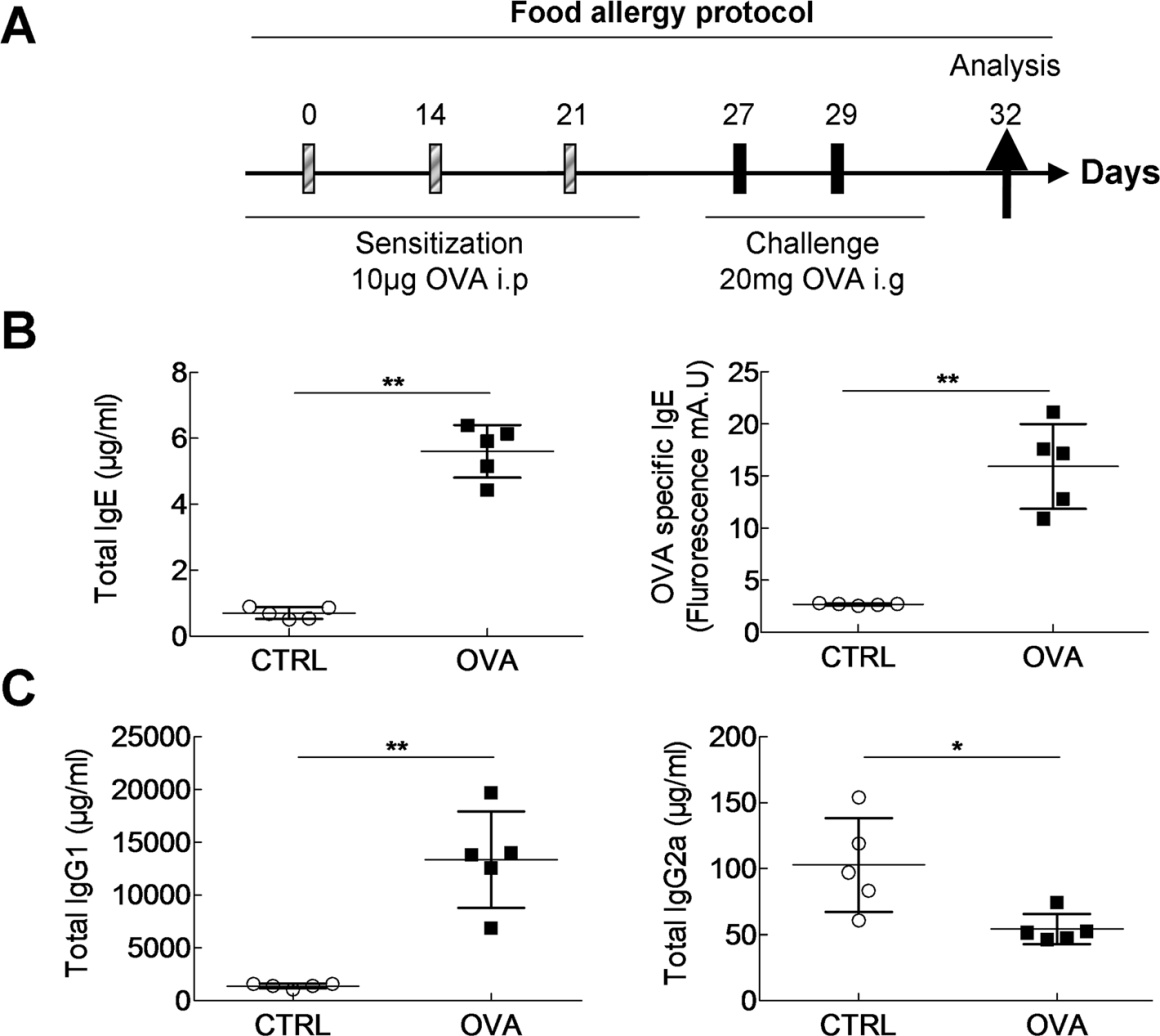
**Mouse model of type I mediated food allergy to Ovalbumin. (A)** Balb/c mice were sensitized three times intra-peritoneally (i.p.) with PBS (CTRL) OVA/Al(OH)_3_ (OVA) every 7 days and subsequently challenged with two doses of PBS or 20 mg OVA by intra-gastric (i.g) gavage. Analyses were done on day 32, three days after the last challenge. At the end of experiment, blood was removed and serum collected to measure **(B)** total IgE, OVA-specific IgE, **(C)** total IgG1 and IgG2a by ELISA in control (white circles) and OVA-allergic mice (black squares). Data are represented as mean ± SEM (n =5 mice/group). *p < 0.05, **p < 0.01.

**Figure 2:**
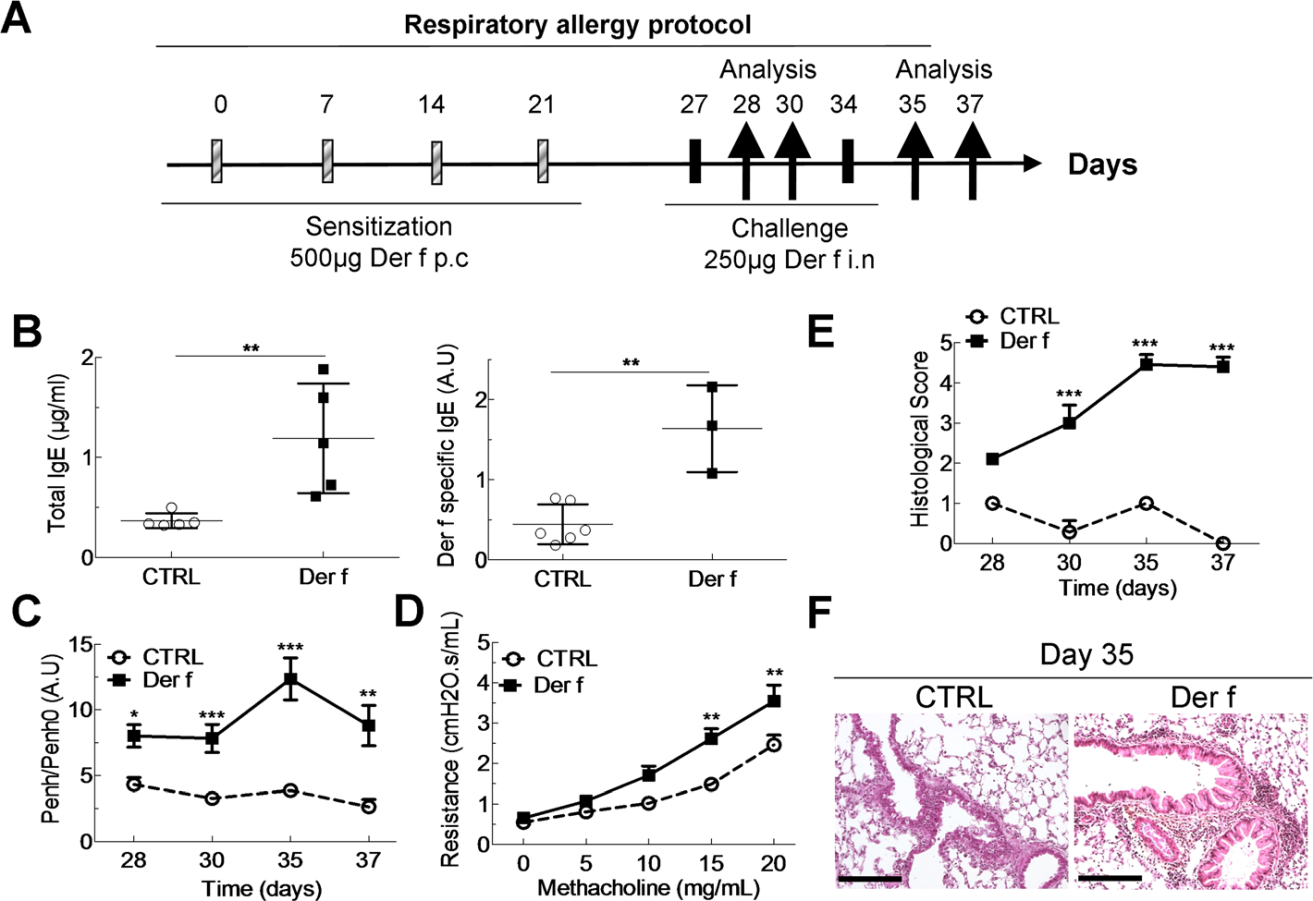
**Mouse model of Der f-induced asthma defined by hyperresponsiveness and pulmonary infiltrate. (A)** Balb/c mice sensitized to Der f by percutaneous (p.c) application on days 0, 7, 14 and 21 and then challenged intranasally (i.n) with Der f on day 27 and 34. Mice were sacrificed after one or three days after the first (day 28 and 30) or second challenge (day 35 and 37). On day 35, blood was removed and serum collected to measure **(B)** total IgE, Der f-specific IgE, by ELISA in control (white circles) and Der f-allergic mice (black squares). **(C)** Measurement of airway hyperresponsiveness (AHR) after one and three days after the first (day 27) and second challenge (day 34) in control (CTRL, white circle dotted line) and in allergic (Der f, black square plain line) mice. AHR is displayed by Penh/Penh0 (pause enhanced ratio to basal pause enhanced). **(D)** Airway resistances to increasing doses of methacholin on day 37 in CTRL and Der-f sensitized mice. **(E)** Inflammatory score in lungs one and three days after the first (day 27) and second challenge (day 34) in CTRL and Der f mice. **(F)** Representative hematoxylin-eosin staining of a lung section in control (CTRL) and allergic mice (Der f) on day 35. Scale bars represent 100 μm. Data are represented as mean ± SEM (n = at least 5 mice/group). *p < 0.05, **p < 0.01, ***p < 0.001.

**Figure 3:**
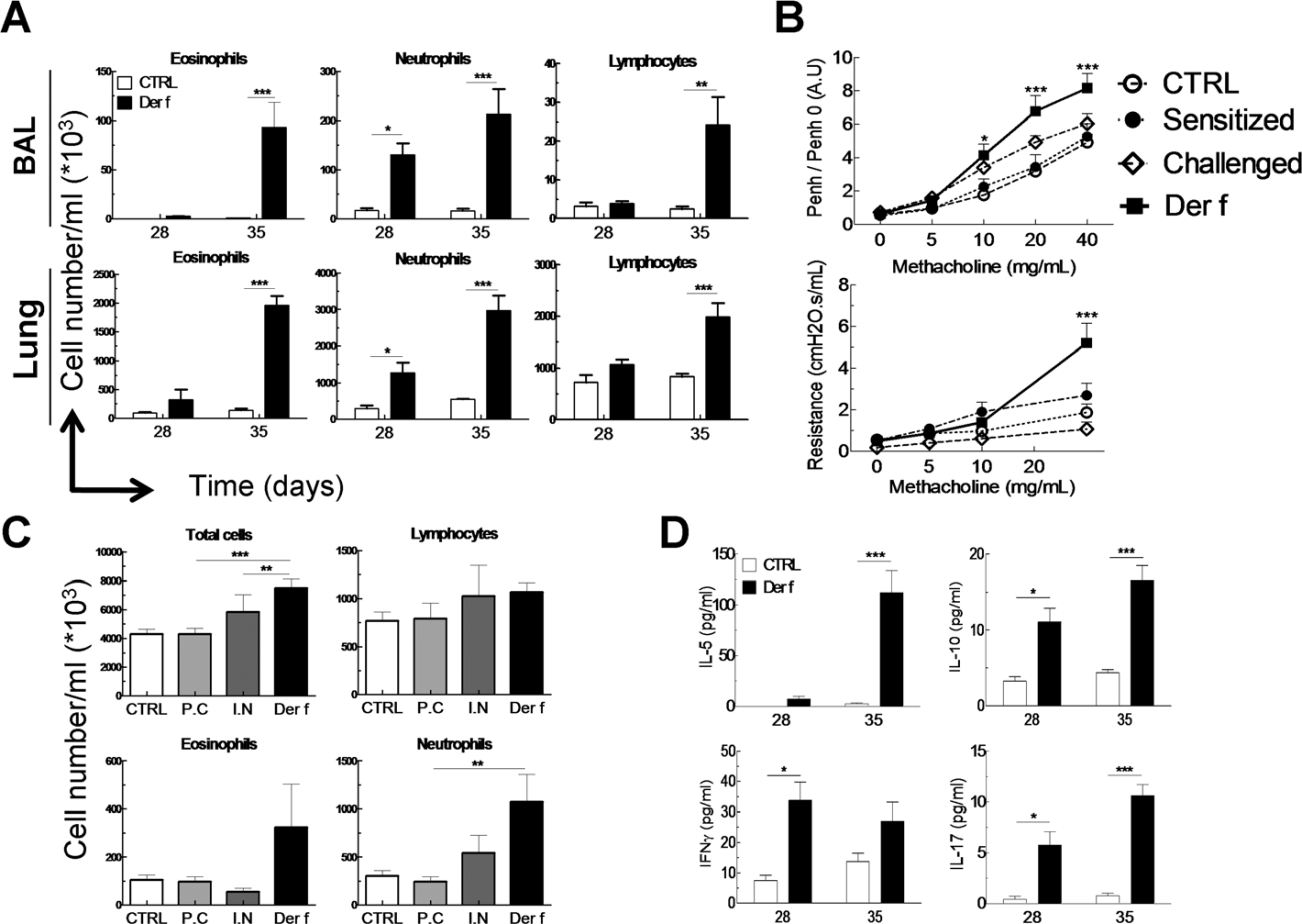
**Influence of exacerbation on lung and BAL inflammation in Der f-induced asthma model. (A)** Eosinophil, neutrophil and lymphocyte counts in BAL and lungs one day after the first (day 28) and second challenge (day 35) in control (white bars) and asthmatic (black bars) mice. **(B)** Measurement of airway hyperresponsiveness (AHR) and airway resistances to increasing doses of methacholin in CTRL (white circles), sensitized (black circles), challenged (white diamonds) and asthmatic mice on day 28. **(C)** Total cells, lymphocytes, eosinophil and neutrophil counts in lungs one day after the first challenge (day 28) in CTRL (white bars), percutaneously sensitized (p.c, light grey), intra-nasally challenged (i.n, dark grey) and asthmatic (Der f, black) mice. **(D)** Measurement of IL-5, IL-10, IFN-γ and IL-17 secretion in BAL cells on day 28 and 35 in CTRL and Der f mice by ELISA. Data are represented as mean ± SEM (n = at least 5 mice/group). *p < 0.05, **p < 0.01, ***p < 0.001.

6 weeks old female Balb/c mice were sensitized for the food allergy, respiratory allergy or both. The induction of the food allergy was carried out on days 0, 14 and 21 by intraperitoneal (i.p.) injection of 10 μg of OVA (Sigma-Aldrich, L'Isle d'Abeau Chesnes, France) diluted in 100 μl of Imject® Alum (Pierce, Rockford, USA) followed by intra-gastric (i.g.) administration of 20 mg of OVA solubilized in water on D27 to D29 (Figure 1A). Control mice were i.p.-sensitized with alum and challenged with water. Respiratory allergy was obtained as follows: mice were sensitized on days 0, 7, 14 and 21 by cutaneous application (p.c.) of 500 μg of total extract of Der f (Stallergenes, Antony, France) diluted in 20 μL of dimethylsulfoxyde (DMSO, Sigma-Aldrich, L'Isle d'Abeau Chesnes, France). Mice were challenged intranasally (i.n.) with 250 μg of Der f on D27 and D34 (Figure 2A) diluted in PBS. Control mice were sensitized with DMSO alone and challenged with PBS. Combined food and respiratory allergy was obtained by applying the two protocols successively (Figure 3A). For respiratory sensitization and challenges animals were anesthetized with 100 μl of i.p. xylazine (15 mg/kg) and 100 μl of i.m. ketamine (80 mg/kg). Mice were sacrificed using dolethal (Vetoquinol). Analyses were performed three days after the food challenge (day 32), after the 1^st^ respiratory challenge (day 28 and 30) and after the 2^nd^ respiratory challenge (day 35 and 37).

### Gastro-intestinal tract monitoring

After 2 hours fast, fecal pellets were collected, counted and weighed. For permeability measurements, the jejunum and proximal colon were removed, washed in cold Krebs’s solution and sections were mounted in Ussing chambers (Physiological instruments, San Diego, CA). Paracellular permeability was assayed by fluorescein–5.6 sulfonic acid (1 mg/mL, Invitrogen) gradient over time by fluorimetry (Varioskan, Thermo SA, France). Transcellular permeability was assayed by HRP (10 mg/ml, Sigma-Aldrich) activity over the time using a Varioskan spectrofluorimeter (Thermo SA, Saint Herblain, France).

### Airway hyperresponsiveness measurement

Unrestrained mice were nebulized in a plethysmography chamber with increasing doses of methacholin (0-40 mg/mL). Airway function was measured and expressed as enhanced pause (Penh) (Emka, Paris, France). Dynamic lung resistances were measured using the flexivent® (SCIREQ, Emka) system. Mice were anesthetized with a mix of xylazine (0.05 mg/kg), ventilated, paralyzed with rocuronium bromide (Organon, 10 mg/ml) and nebulized with methacholin (0-20 mg/mL). Airway resistances were continuously monitored and recorded according to manufacturer’s instructions.

### Histology

1 mL of paraformaldehyde (PFA) 4% was administered intra-tracheally. Excised lungs were fixed in PFA 4%, embedded in paraffin, cut and stained with hematoxylin and eosin for morphological study and inflammation scoring from 0 to 7 representing bronchial epithelial cell dystrophy grades from 0 to 3 and peribronchial/perivascular inflammatory infiltrate abundance grades from 0 to 4.

### Immunoglobulin assays

Total IgE and IgG1 were assayed by ELISA in serum. Specific anti-Der f 1 IgE and anti-OVA IgE were assessed by indirect ELISA. 96 wells plates were coated with sodium bicarbonate 50 μM and 0.25 μg of purified Der f 1 (Indoor Biotechnologies, Wiltshire, UK) or 5 μg OVA (Sigma-Aldrich, L'Isle d'Abeau Chesnes, France) and left overnight at 4°C. Wells were then incubated with bovine serum albumine 1% for 12 h at 4°C, washed, filled with sera diluted in CGS1 (Cosmo Bio, California, USA) and incubated overnight at 4°C. Specific Ig was revealed with anti-mouse IgE (AbD serotec, Colmar, France) coupled to HRP. Substrate ABTS (Roche, Boulogne-Billancourt, France) was added and absorbance measured at 405 nm with VictorTMX3 (PerkinElmer, Courtaboeuf, France).

### Flow cytometry in broncho-alveolar lavages and lungs

Broncho-alveolar lavage (BAL) fluid was recovered by intra-tracheal administration and aspiration of 1 mL of PBS. To obtain total lung immune cells, the lungs were disrupted and digested at 37°C for 1H in a solution containing Dnase I (Roche) and collagenase II (Invitrogen). To determine the immune profile, lung and BAL cells were counted and stained by flow cytometry as previously described [16] with specific anti-mouse antibodies: CD3-APC, CD8-APC-H7, CD19 PE-Cy7, F4/80-FITC, Ly6G-PerCP-Cy5.5, and CCR3-PE (R&D, Lille, France). Analyses were performed on a BD LSR™ II with BD FACSDiva™ software (BD Biosciences).

### Cytokine quantification in broncho-alveolar lavage

Cytokines (IL-4, IL-5, IL-10, IFN-γ, IL-17, chemoattractant keratinocyte (KC) and CCL5/RANTES (Regulation on Activation, Normal T cell Expressed and Secreted) were quantified by Luminex (BioPlex 200 system, Bio-Rad, Marnes-La-Coquette, France) according to manufacturer’s instructions (Bio-Rad).

### Statistics

GraphPad Prism 4.0b (Graphpad Software Inc., La Jolla, CA USA) was used. Data are expressed as mean ± standard error to the mean (SEM). Means were compared among groups using ANOVA. In case of positive ANOVA means were compared by Mann-Whitney test. Results were corrected by a Bonferroni multiple comparisons correction test. A p < 0.05 was considered statistically significant.

## Results

### OVA i.p. sensitization and oral challenge induce type I food allergy and increase gut permeability

To characterize the food allergy part of the model, serum-allergen specificity and intestinal function of OVA and control mice were evaluated on day 32, 3 days after final challenge (Figure 1A). Type I allergy was confirmed by a significant increase of total (697 vs 5603 ng/ml) and OVA-specific IgE levels (2680 vs 15913 fluorescence units) in OVA mice compared to controls (Figure 1B). Th2-supported IgG1, and Th1-supported IgG2a proteoglycan-specific antibody levels in serum were determined. The IgG2a predominance (1.33.10^7^ vs 1,399.10^6^ ng/ml) in allergic mice suggests a bias towards a Th2-type response (Figure 1C). Intestinal parameters were assessed 30 minutes after the final intra-gastric challenge (day 29) (Table 1). Transit time, fresh faecal pellet weight and humidity were similar in both groups. Contrastingly, a significant increase in paracellular permeability was found in OVA mice, whereas there was no difference is transcellular permeability between the groups. OVA sensitization did not trigger any change in respiratory function or airway inflammation (not shown). Taken together these results show that OVA i.p. sensitization and intra-gastric challenge induces a systemic type I allergy and an increase in intestinal permeability.

**Table 1:** **OVA-induced food allergy model induces minor alterations on intestinal parameters**

	**CTRL**	**OVA**
Total transit time (mn)	185,10 ± 39,84	188,25 ± 54,31
Total fresh fecal pellet weight (mg)	145,67 ± 45,41	129,90 ± 56,73
% humidity of fecal pellet	73,65 ± 2,62	73,32 ± 3,25
Paracellular in vivo permeability (Fluo (AU)/μl plasma)	6,12 ± 1,60	7,73 ± 2,88*
Transcellular *in vivo* permeability (ng/ml)	403,60 ± 150,89	503,05 ± 268,98
Paracellular *ex-vivo* permeability in jejunum (sulfonic acid flux (slope))	0,60 ± 0,34	1,43 ± 0,15*
Paracellular *ex-vivo* permeability in colon (sulfonic acid flux (slope))	0,23 ± 0,04	0,18 ± 0,02

Intestinal parameters including transit time, pellet weight and humidity and permeability were measured 30 minutes after the last challenge with OVA on day 29 in control (CTRL) and in OVA-induced food allergy (OVA) mice. *p<0.05.

### Der f skin sensitization and intranasal challenge induce type 1 respiratory allergy with increased airways resistances and lung inflammation

In order to follow as closely as possible the atopic march, asthma was induced by percutaneous sensitization and intranasal challenge (Figure 2A). Total and specific serum IgE levels significantly increased in Der f-allergic mice on day 35, one day after the 2^nd^ challenge (0.36 vs 1.14 μg/ml and 0.44 vs 1.6 A.U respectively) (Figure 2B), but not on day 26, before challenges (not shown), suggesting that skin exposure is not sufficient for inducing a full allergic response and that bronchial exposure is essential. Regarding respiratory function, Der f-allergic mice displayed airway hyperresponsiveness (AHR) one and three days (day 28 and day 30) after the first challenge (Figure 2C). A second challenge on day 35 increased AHR dramatically, a result confirmed using flexivent® on day 37 (Figure 2D). Overall tissue inflammation was assessed by histological evaluation of lung sections at the same time points. A clear increase in inflammation between the 1^st^ (day 28) and the 2^nd^ challenge (day 35) (Figure 2E) was observed, concordant with respiratory function measurements. Figure 2F shows the extensive perivascular and peribronchial cell infiltration on day 35 (Figure 2F).

In order to characterize the inflammatory profile induced by Der f in the model, the proportions of eosinophils, neutrophils and lymphocytes in lungs and BAL were analyzed after the 1^st^ (day 28) and the 2^nd^ challenge (day 35) (Figure 3). On day 28, a significant increase in neutrophils (16.10^3^*versus* 130.10^3^ cells), but not in eosinophils and lymphocytes was identified in BAL (Figure 3A). These results were confirmed in lungs, with a significant increase of total cell concentration, due to a major influx of neutrophils (287.10^3^ vs 1275.10^3^ cells) and to a lesser extent of eosinophils and lymphocytes (Figure 3A). After the second challenge (day 35) proportions of eosinophils, neutrophils and lymphocytes were strongly elevated in BAL and lungs from allergic mice compared to control mice and mice which had only undergone one challenge (Figure 3A).

In order to assess the role of skin sensitization in the asthma process, experiments were also performed in challenged, unsensitized mice and in sensitized, unchallenged mice. In neither group difference in AHR was found compared to control mice (Figure 3B). In addition, sensitization or challenge alone did not induce inflammation, detected by cell infiltration in the lungs (Figure 3C). These results show that skin sensitization to HDM is necessary, but not sufficient, to induce asthma. In order to further define the mechanisms of allergen-induced inflammation, cytokines specific to Th1 (IFN-γ), Th2 (IL-5), Th17 (IL-17) and Treg (IL-10) activation were assayed in BAL. IFN-γ, IL-17 and IL-10 significantly increased in BAL after the 1^st^ challenge, whereas IL-5 significantly increased only after the 2^nd^ (Figure 3D). Interestingly, secretion of IL-5, IL-10 and IL-17 increased greatly after the 2^nd^ challenge (Day 35). Similar results were observed in lungs (data not shown). These data highlight a Th1/Th17-oriented inflammation after the first challenge and a mixed Th2/Th17 inflammation after the second challenge.

Overall, these results show that cutaneous sensitization with Der f followed by Der f respiratory challenges induces a systemic type one allergy to Der f. Skin sensitization primes the immune system to respond to a pulmonary challenge with a biphasic lung inflammation and hugely increased AHR after a second challenge.

### Prior OVA-induced food allergy affects immunoglobulin levels, respiratory function and inflammation in Der p-induced asthma.

**Figure 4:**
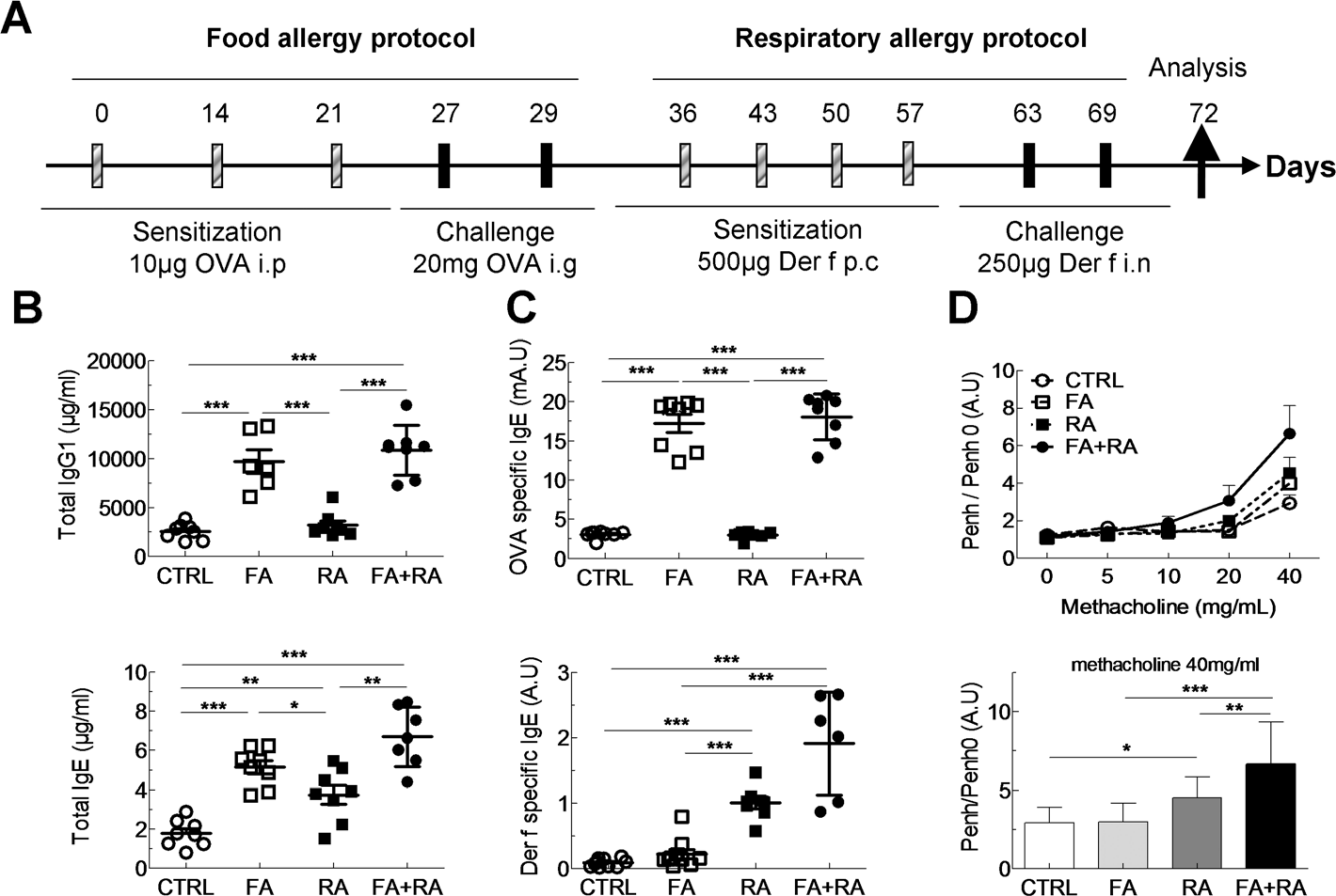
**OVA-induced gastrointestinal food allergy increases immunoglobulin production and allergic airways response in Der f-induced asthma model. (A)** An OVA food allergy followed by respiratory allergy were induced in Balb/c mice. At the end of the protocol, blood was removed and serum collected to quantify **(B)** total IgG1 and IgE as well as **(C)** Der f-specific and OVA-specific IgE by ELISA in controls (CTRL, white circles), OVA-induced food allergy (FA, white squares), Der f-induced asthma (RA, black squares) and bi-allergic Der f-induced asthma with OVA-induced food allergy (FA + RA, black circles) mice. **(D)** Measurement of AHR with increasing doses of methacholin (upper panel) and to the higher dose (lower panel) in control (white circles), FA (white squares), RA (black squares) and bi-allergic FA + RA (black circles) mice. Data are represented as mean ± SEM (n = at least 5 mice/group). *p < 0.05, **p < 0.01, ***p < 0.001.

In order to assess whether a synergy between OVA and HDM allergies could aggravate asthma in food allergic mice, a number of parameters were compared between respiratory allergy, food allergy and dual (food plus respiratory) allergy. Immunoglobulin levels were assayed in serum obtained 3 days after the last intranasal HDM challenge. Total IgG1 and IgE (Figure 4B) levels were significantly higher in dual allergy mice compared to control and mice with asthma only. However total IgG1 levels were not higher in dual allergy mice than in mice allergic to OVA only. As expected, OVA-specific IgE levels were significantly higher in food allergic mice. In dual allergy mice, no further increase in OVA-specific IgE was observed (Figure 4C, upper panel).

**Figure 5:**
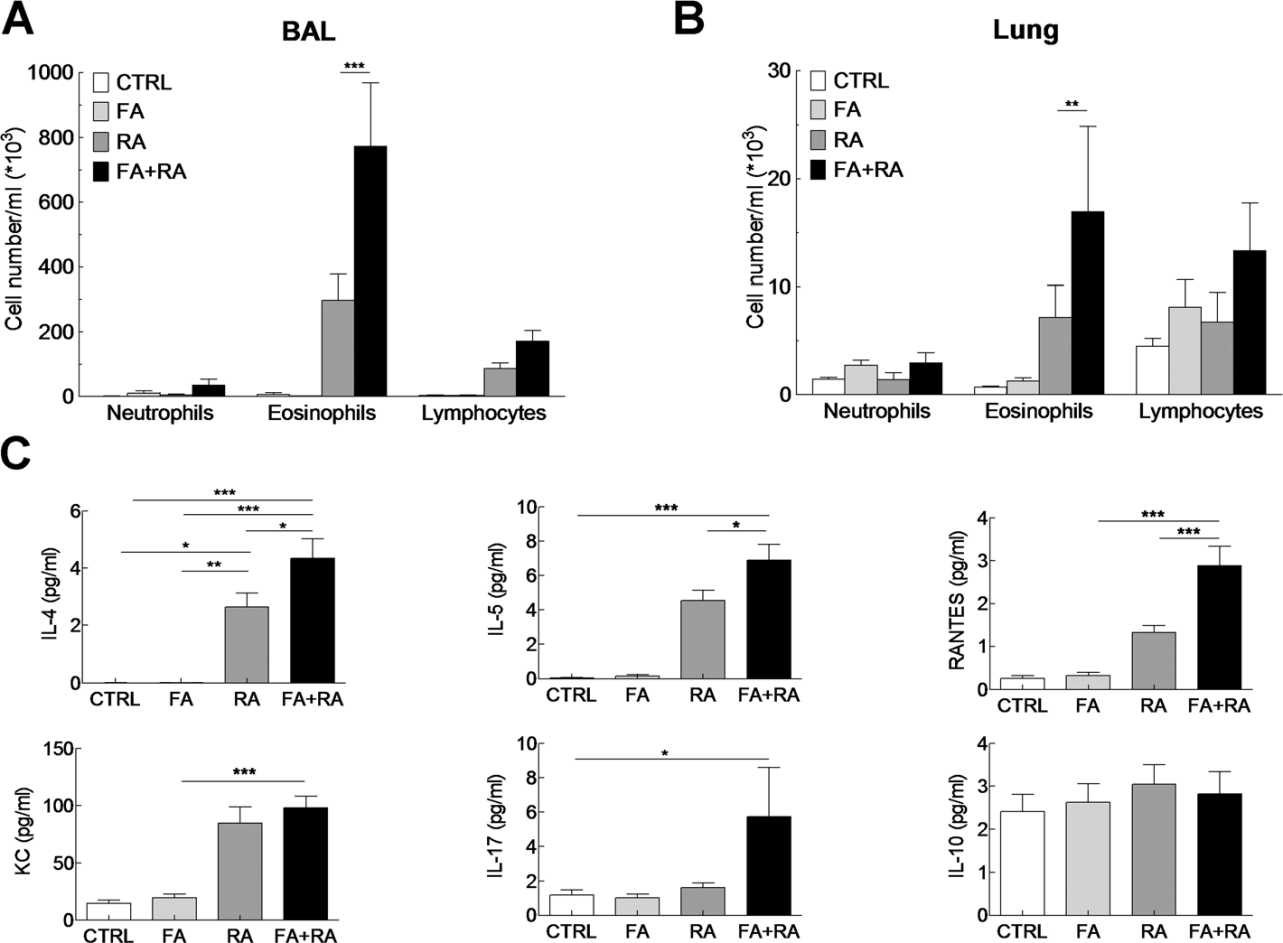
**OVA-induced gastrointestinal food allergy influences pulmonary inflammation and cytokine production in Der f-induced asthma model.** Eosinophils, neutrophils and lymphocytes count in **(A)** BAL and in **(B)** lungs in control (white bars), FA (light grey bars), RA (dark grey squares) and bi-allergic FA + RA (black bars) mice. **(C)** Measurement of IL-4, IL-5, IL-10, IL-17, KC and RANTES secretion in BAL cells by ELISA in the different groups as in A. Data are represented as mean ± SEM (n = at least 5 mice/group). *p < 0.05, **p < 0.01, ***p < 0.001.

In contrast, Der f-specific IgE levels were higher in asthmatic mice and significantly higher in dual allergy mice compared to control and mice allergic to OVA only (Figure 4C, lower panel). Dual allergy mice displayed significantly greater AHR than mice sensitized to one allergen only (Figure 4D). Inflammation was also increased in BAL and lungs of mice with two allergies (Figure 5A and B, respectively). Neutrophils, eosinophils and lymphocytes numbers were elevated in dual allergy mice compared to all other groups, with a predominant eosinophil infiltrate. Cytokine production was analysed in BAL supernatants (Figure 5C). As expected from the inflammatory profile, BAL IL-4 and IL-5 levels were increased in asthmatic mice compared to controls and mice allergic to OVA only. BAL IL-4 and IL-5 were also found increased in dual allergy mice compared to asthmatic, OVA allergic and controls mice. In addition RANTES, a chemotactic agent for T cells, eosinophils and basophils, was significantly higher in dual allergy mice than in others groups. However, KC, a chemotactic agent for neutrophils, was found at significantly higher levels in asthmatic mice, whether they were also OVA-allergic or not, than in controls and mice only allergic to OVA. Similarly, IL-17 was significantly increased in dual allergy mice compared to controls, but not compared to mice allergic to a single agent. IL-10 was found at the same level in all groups (Figure 5C).

These results show that an OVA food allergy primes the immune system to increase its response to Der f respiratory challenges in mice percutaneously sensitized to Der f.

## Discussion

In this study, we describe a dual allergy mouse model with an OVA-induced food allergy followed by asthma obtained by sensitization to Der f extract. The OVA allergy was induced with 3 intra-peritoneal sensitizations and 2 intra-gastric challenges. Sensitization was effective, as reflected by the increase in total and OVA-specific IgE. Yet, mice displayed no symptom of food allergy, although *in-vivo* and *ex-vivo* permeability was increased. These results contrast with those obtained by Zhang *et a*l., [17], who reported severe diarrhea in allergy induced by i.p. injection of 20 μg and intra-gastric challenges of 100 μg of OVA. Similarly, Perrier *et al.,* [18] observed decreased body temperature, pruritus and diarrhea in response to intra-gastric sensitizations and challenges with OVA. These discrepancies could be explained by the fact that we did not use any adjuvant such as cholera toxin [18] or Alum [17] during challenges. The sensitization to OVA was, however, sufficient to facilitate the development of a subsequent systemic and respiratory response to skin and lung Der f exposure. It is noteworthy that a similar scenario occurs in human subjects in whom sensitization to eggs (presence of specific IgE, positive skin tests) is frequent in atopic subjects who can however eat eggs without any symptoms.

In order to induce the respiratory allergy, mice were sensitized to HDM through application of Der f extract on the ear, mimicking HDM sensitization reported in atopic infants [11]. Repeated skin applications of Der f would induce eczema [19] but they were limited so that no lesion occurred. This also reflects the frequent asymptomatic sensitization to HDM seen in atopic subjects. The effectiveness of cutaneous sensitization was shown by the 1^st^ challenge, which induced inflammation and AHR in sensitized mice only. HDM challenges induced, in turn, an increased bronchial response to methacholin with an increase in inflammation together with a rise in total and Der f-specific IgE. In addition, lung histology showed perivascular and peribronchial infiltrates. This pattern of pulmonary inflammation is highly relevant to the human situation and was confirmed by a sequential influx of neutrophils and eosinophils in the lungs, thus reinforcing the relevance of the model, in particular for severe human cases [20] in which a mixed inflammation with a consistent neutrophil infiltrate accompanying the eosinophilic contingent is frequently found.

Asthma was more pronounced in mice previously sensitized to food with total and specific IgE levels being higher in these mice compared to all other groups. Most significantly, AHR was clearly higher in bi-allergic compared to mono-allergic mice. This asthma outcome occurred in a context of increased inflammation in lungs and BAL. Moreover the presence of a food allergy induced further BAL IL-4 and IL-5 increase in HDM-sensitized mice demonstrating a Th2 response enhancement. Accordingly eosinophil numbers and RANTES production were dramatically higher in bi-allergic mice compared to their mono-allergic counterparts [21].

**Figure 6:**
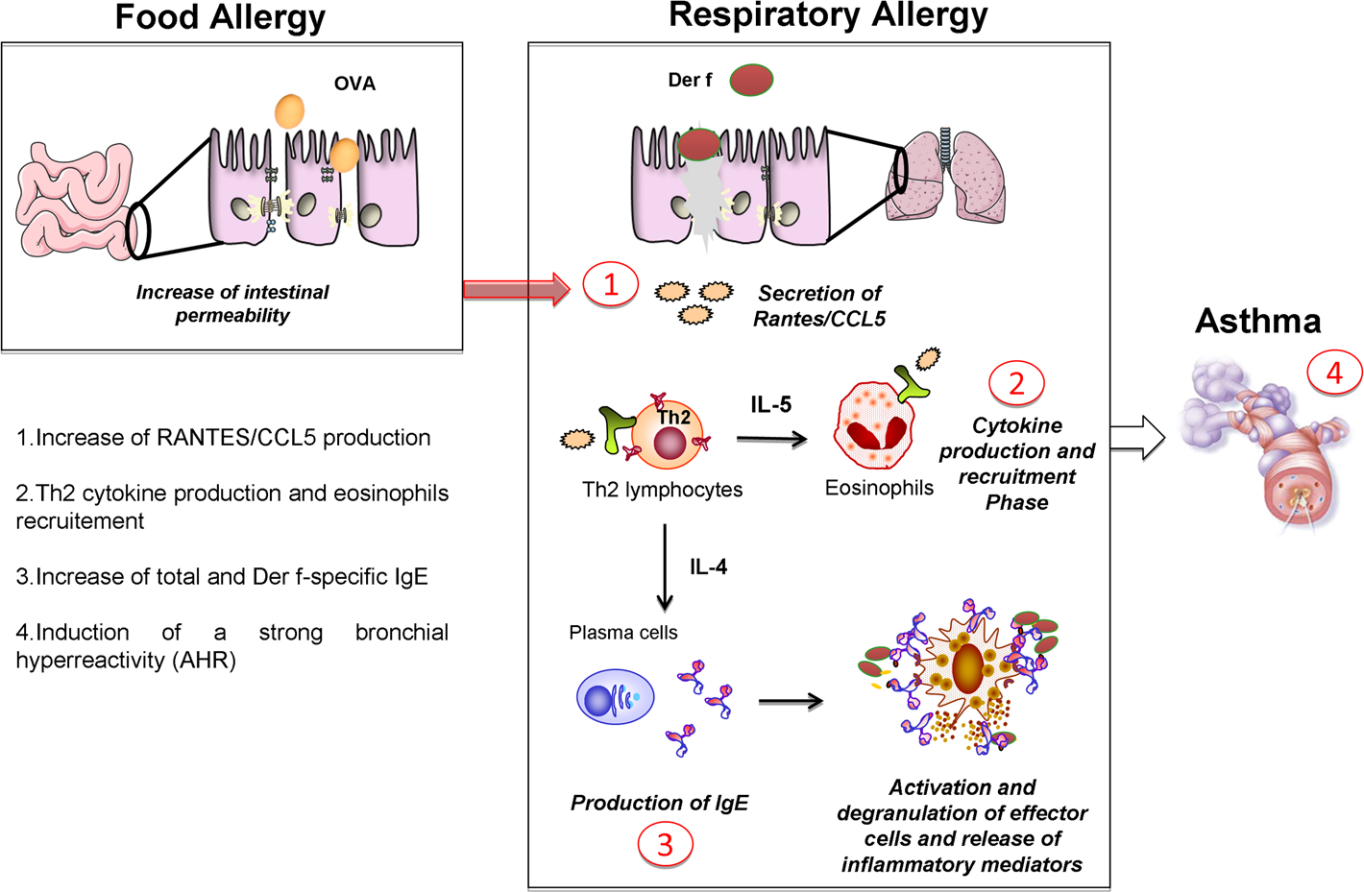
**Hypothesis of the influence of food allergy on the development of asthma.** Initial sensitization to food allergen will lead to allergic reaction after allergen re-exposure and induce an increase of intestinal permeability. Then, the development of asthma is influenced by the previous food allergy and this influence is characterized by an increase of RANTES/CCL5 production **(1)** as well as a stronger activation and recruitment of Th2 cells and eosinophils **(2)**. These events will result in an increase of total and Der f-specific IgE **(3)** to induce activation and degranulation of inflammatory cells, leading to the induction of a strong bronchial hyperresponsiveness (AHR) and to severe asthma **(4)**.

Brandt *et al.,* [22] have previously shown that gastro-intestinal allergy to OVA increases AHR, hypereosinophilia and global hypercellularity in response to subsequent HDM challenges. However this result was obtained after three challenges and a fourth challenge was necessary to induce specific IgG1 production. In addition no significant pulmonary inflammation was obtained in non-OVA-sensitized animals. In our study, the initial trans-cutaneous sensitization explains why we obtained a significant asthmatic reaction in ovalbumin-naïve mice and a dramatically enhanced pulmonary inflammation in response to HDM after only 2 challenges in OVA-sensitized animals. Our model therefore clearly highlights the importance of the three allergen-impacted compartments, gut, skin and lungs, and the synergy between their respective immune responses in the development of asthma (Figure 6).

Our results demonstrate that a primary sensitization to one allergen primes the immune system to develop an intense response to a subsequently administered fully dissimilar inhaled allergen. This sequence shows that, rather than being independent events occurring in response to multiple exposures on a common genetic background, the sensitization to multiple allergens observed in atopic subjects may result from a synergic interaction between the immune responses initiated by each allergen. These results are concordant with epidemiological data demonstrating that sensitization to food, and notably to eggs, is an important risk factor for sensitization to inhaled allergens, atopic dermatitis and asthma [23].

## Conclusion

In conclusion our findings provide a model that could be instrumental for further elucidating the mechanisms of atopic march. It will be useful to find new prophylactic strategies to prevent asthma in children with food allergies and/or atopic dermatitis and to test new drugs in preclinical settings. The full mechanisms involved have still to be deciphered.

## Abbreviations

AHR: Airway hyperresponsiveness
BAL: Bronchoalveolar lavage
Der f: Dermatophagoides Farinae
FEV1: Forced expiratory volume in 1 second
HDM: House dust mite
HRP: horseradish peroxidase
IgE: Immunoglobulin E
I.M: intra-muscular administration
I.P: Intra-Peritoneal
MCh: Methacholin
OVA: Ovalbumin
PBS: Phosphate buffer saline
Penh: Enhanced pause
P.C: Percutaneous
